# The incidence of tuberculosis among hiv-positive individuals with high CD4 counts: implications for policy

**DOI:** 10.1186/s12879-016-1598-8

**Published:** 2016-06-10

**Authors:** Tendesayi Kufa, Violet Chihota, Victor Mngomezulu, Salome Charalambous, Suzanne Verver, Gavin Churchyard, Martien Borgdorff

**Affiliations:** The Aurum Institute, 29 Queens Road, Parktown, Johannesburg, 2193 South Africa; The School of Public Health, University of the Witwatersrand, Johannesburg, South Africa; Department of Diagnostic Radiology, University of the Witwatersrand, Johannesburg, South Africa; KNCV Tuberculosis Foundation, The Hague, The Netherlands; Academic Medical Centre, University of Amsterdam, Amsterdam, The Netherlands

**Keywords:** Antiretroviral, Therapy, Intensified, Case, Finding

## Abstract

**Background:**

Intensified case finding (ICF) and earlier antiretroviral therapy (ART) initiation are strategies to reduce burden of HIV-associated tuberculosis (TB). We describe incidence of and associated factors for TB among HIV-positive individuals with CD4 counts > 350 cells/μl in South Africa.

**Methods:**

Prospective cohort study of individuals recruited for a TB vaccine trial. Eligible individuals without prevalent TB were followed up at 6 and 12 months after enrolment. Cox proportional hazards regression was used to determine factors associated with risk of incident TB.

**Results:**

Six hundred thirty-four individuals were included in the analysis [80.9 % female, 57.9 % on ART, median CD4 count 562 cells/μl (IQR 466–694 cells/μl)]. TB incidence was 2.7 per 100 person-years (pyrs) (95 % CI 1.6–4.4 per 100 pyrs) and did not differ significantly between those on ART and those not on ART [HR 0.65 (95 % CI 0.24–1.81)]. Low body mass index (BMI <18.5 kg/m^2^) was associated with incident TB [HR 3.87 (95 % CI 1.09–13.73)]. Half of the cases occurred in the first 6 months of follow up and may have been prevalent or incubating cases at enrolment.

**Conclusions:**

TB incidence was high and associated with low BMI. Intensified case finding for TB should be strengthened for all HIV positive individuals regardless of their CD4 count or ART status.

**Electronic supplementary material:**

The online version of this article (doi:10.1186/s12879-016-1598-8) contains supplementary material, which is available to authorized users.

## Background

Tuberculosis (TB) remains a public health concern in high HIV prevalence settings. In 2013, the World Health Organisation (WHO) estimated that in South Africa, 62 % of individuals newly diagnosed with TB were HIV positive [1]. Failure to address HIV-associated TB will result in failure to achieve the global targets for TB control or elimination [2].

The increase in the risk of TB associated with HIV occurs early in the course of HIV infection and increases rapidly as the CD4 count decreases [3–5]. The initiation of antiretroviral therapy (ART) in HIV-positive individuals should theoretically decrease the risk of TB regardless of CD4 count. The restoration of TB specific immunity with ART use is known to be incomplete and variable such that HIV-positive individuals on ART remain at elevated risk of TB compared to HIV negative persons. Restoration in TB specific immunity has been shown to be poorer among individuals with lower nadir CD4 counts [6, 7]. Initiating ART at higher CD4 counts (>350cells/μl) has been shown to reduce TB incidence [8, 9]. South Africa recently announced an increase in the cut-off for ART initiation to 500 cells/μl, [10] largely for its benefits in reducing HIV- related morbidity and HIV transmission [11]. The latest guidance moved the country towards implementation of the universal test and treat approach, which will see all HIV-positive individuals being eligible for ART regardless of CD4 count. The objective of this study was to assess the incidence of TB in a cohort of HIV positive individuals with CD4 counts ≥350 cells/μl, assess factors associated with risk of incident TB and discuss the implications for TB prevention. The study was originally set-up to determine baseline incidence for future TB vaccine trials among HIV- positive persons, but could also provide useful information for programme strategies.

## Methods

### Location and setting

Between June 2011 and June 2012, consecutive HIV-positive adults attending care at two primary care facilities in Gauteng Province, South Africa were invited to participate in a TB vaccine preparedness study including HIV- positive individuals with a recent CD4 counts > 300cells/μl. This cut-off was selected to match the inclusion criteria for TB vaccine trials including HIV-positive individuals at the time. In this secondary analysis we present data on the incidence of TB among a subset of participants with confirmed CD4 counts > 350 cells/μl. The anticipated TB vaccine trial intended to enrol individuals who were aged between 18 and 45 years of age, had a documented HIV positive result with CD4 count > 350 cells/μl, were healthy and independently able to carry out activities of daily living, willing and able to avoid pregnancy or elective surgery during the trial and able to complete study procedures and follow up. Additional criteria for inclusion in the vaccine preparedness study were : i) having a documented CD4 count > 300cells/μl in the 12 months preceding enrolment regardless of whether or not they were taking ART, ii) and be living or working in the communities surrounding the clinics.

### Study population and procedures

Study- specific recruiters identified individuals as they registered for HIV care and referred them to a study nurse located in a different part of the clinic for assessment of eligibility to enrol in the study. The study nurse enrolled eligible and consenting individuals and administered a questionnaire collecting data on demographic characteristics, history of previous exposure to or treatment for TB, history of HIV care and treatment (including ART) and presence of symptoms suggestive of TB [cough, fever, night sweats or weight loss of any duration]. Participants had their heights and weights measured and were asked to provide a blood specimen for CD4 count testing. Female participants were asked to provide a urine specimen in order to exclude pregnancy. Participants who reported symptoms suggestive of TB were also asked to provide two sputum specimens for smear microscopy and mycobacterial culture and had a chest radiograph done in order to exclude TB at enrolment (see Additional files 1, 2, 3 and 4). The chest radiographs were sent to a single radiologist who read them using a standardized tool and determined if the chest radiograph findings were likely to be a result of active TB (see Additional file 4).

As this study was planned to estimate TB incidence among participants who would be eligible for a TB vaccine trial, participants who were pregnant, were on TB treatment, or diagnosed with TB at enrolment or whose enrolment culture results came back positive were not eligible for inclusion and follow-up. Those eligible for follow up were invited to return for study visits at 6 and 12 months after enrolment. At each follow up visit, the study nurse administered a questionnaire enquiring about TB diagnoses, ART, isoniazid preventive therapy (IPT), cotrimoxazole preventive therapy (CPT) initiation and symptoms suggestive of TB. If at follow up, participants reported symptoms suggestive of TB, they had sputum specimens collected for smear microscopy and culture and sent for a chest radiograph to exclude TB (see Additional files 5 and 6). Participants who missed follow up visits were contacted by telephone and requested to come for follow up. If the participants could not be reached, their next of kin were contacted using contact details provided by participants at enrolment. At the next of kin contact, the study team enquired about vital status and if alive about alternative contact details of the participant. The study team also independently reviewed the HIV clinic records of all participants and abstracted data on TB and HIV care in order to verify some self- reported data. Completed study questionnaires were couriered to a central data management centre and data entered into a study specific database. Data were exported into STATA® 12.0 (College Station, Texas, USA) for analysis. Study follow up procedures were conducted until March 2013 (see Additional files 7 and 8).

### Data analysis

Participants who were pregnant, taking TB treatment, or had TB diagnosed at enrolment or within the first 3 months of enrolment were excluded from the secondary analysis as were those who had CD4 counts < 350 cells/μl. Participants included were described using medians and interquartile ranges for continuous variables and proportions for categorical variables, comparing those on ART with those not on ART at enrolment. Follow-up time (person-years of follow-up) was determined from date of enrolment until diagnosis of TB, death, or date of last study visit, whichever occurred first. Incident TB was defined as having a documented TB treatment initiation by the clinic or having a positive sputum smear or culture or a chest radiograph consistent with active TB at a study follow up visit. The TB incidence estimates were determined and compared for participants on ART with those not on ART. Participants who initiated ART during follow up contributed person- time to both on ART and not on ART groups. Univariable and multivariable Cox proportional hazards regression were used to determine factors associated with incident TB in follow up. Because of the small number of outcomes a maximum of two variables with the smallest p-values under 0.2 in univariable analyses were included the multivariable model with being on ART included a priori. A subgroup analysis excluding participants who reported TB symptoms at enrolment was conducted in order to determine effect of TB screening on incident TB.

## Results

### Study population

**Fig. 1:**
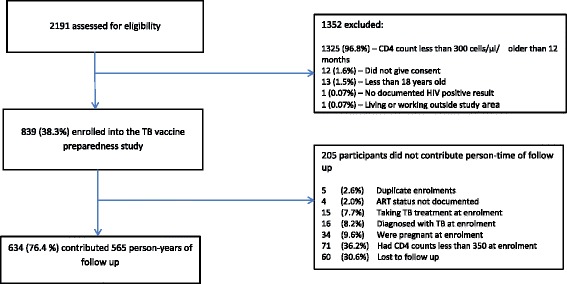
Study flow

Of 2191 participants assessed for eligibility to enrol, 839 were enrolled and 634 were included in this secondary analysis (see Fig. 1). Of the 1352 not eligible for enrolment, the majority were excluded because of low CD4 counts at eligibility assessments. Among those enrolled, a further 205 were excluded from analysis because of duplicate enrolments (*n* = 5), undocumented ART status at enrolment (*n* = 4), taking TB treatment at enrolment (*n* = 15), being diagnosed with TB at enrolment or in the first 3 months of follow up (*n* = 16), pregnant at enrolment (*n* = 34), having CD4 counts <350 cells/μl (*n* = 71) and 60 did not return for any follow up visits and could not be traced.

Among the 634 participants included in the analysis, 19.1 % were male, median age was 36.1 years (IQR 29.8–42.1), 38 (6.0 %) had BMI < 18.5 kg/m^2^, median CD4 count was 562 cells/μl (IQR 466–694) and 367 (57.9 %) had been taking ART for a median of 2.0 years (IQR 1.0–3.9 years) at enrolment. Participants on ART were slightly older [37.4 years (IQR 31.4–42.3 years) vs. 33.5 years (IQR 28.8–40.8 years) *p* < 0.002], more likely to have been treated for TB in the past [31.3 % vs. 19.5 %, *p* < 0.001] but less likely to report current or prior IPT use at enrolment [13.9 % vs. 35.6 %, *p* < 0.001] (Table 1).

**Table 1:** Characteristics of cohort at enrolment (*N* = 634)

Characteristic	All	ART (*n* = 367)	not on ART (267)
Males (n, %)	121 (19.1)	67 (18.3)	54 (20.2)
Age in years (median, IQR)	36.1 (29.8–42.1)	37.4 (31.4–42.3)	33 (28.8–40.8)
Employment (n, %)	254 (40.1)	156 (42.5)	98 (36.7)
Ever smoked (n, %)	143 (22.3)	90 (24.5)	53 (19.8)
Currently drink alcohol (n, %)	251(41.4)	152 (41.4)	99 (37.1)
Duration since HIV test years^€^ (median, IQR)	1.8 (0.8–4.4)	2.5 (1.4–4.9)	1.1 (0.4–2.4)
Duration on ART years (median, IQR)	-	2.0 (1–3.9)	-
Current or prior INH use (n, %)	146 (23)	51 (13.9)	95 (35.6)
Current CPT use (n, %)	94 (14.8)	36 (9.8)	58 (21.7)
Previous TB treatment (n, %)	167 (26.3)	115 (31.3)	52 (19.5)
TB symptoms at enrolment (n, %)	152 (24)	76 (20.7)	76 (28.5)
CD4 count at enrolment (median, IQR)^β^	562 (466–694)	546 (466–688)	575 (456–716)
CD4 counts > 500 cells/μl (n, %)	383 (60.4)	224 (61)	159 (60)
BMI (median, IQR)^α^	24.4 (21.3–27.8)	24.5 (20.1–27.1)	24.5 (21.5–28.4)

€- available for 446 participants (253 on ART and 193 not on ART), β-available for 588 participants (342 on ART and 246 not on ART), α- available for 621 participants (356 on ART and 265 not on ART

### Incidence of TB during follow up

The 634 participants included in the analysis were followed up for a median of 11.2 months (IQR 8.8 months–12.4 months) and contributed 565 person-years at risk during follow up. Due to budgetary constraints, follow up had to be limited to 8 months post enrolment for last participant enrolled. During the follow up period, 15 participants were diagnosed with TB, three died and 18 who were not on ART at enrolment initiated ART. Among the 15 individuals who had incident TB, 10 had pulmonary TB [four smear positive, four were smear negative, culture positive, two were smear negative diagnosed by chest radiograph], one extra-pulmonary TB and remaining four were self-reported by participants but site of disease and smear status not verified with clinic records. The median time to TB diagnosis was 6 months (IQR 3.3–8.3 months). The overall TB incidence rate was 2.7 per 100 person-years (pyrs) (95 % CI 1.6–4.4 per 100 pyrs) and did not differ between participants on ART and those not on ART [2.2 per 100 pyrs (95 % CI 1.0–4.5 per 100 pyrs) vs. 3.4 per 100 pyrs (95 % CI 1.6–7.0 per 100 pyrs)] (see Table 2). Among 224 participants who were on ART and had CD4 counts > 500 cells/μl at enrolment, there were five incident TB cases in 208 pyrs of follow up giving an incidence rate 2.4 per 100 pyrs (95 % CI 1.0–5.8 per 100 pyrs) while among the 159 participants not on ART at enrolment, there were three TB cases in 127 pyrs giving also an incidence of 2.4 per 100pyrs (95 % CI 0.8- 7.3 per 100 pyrs).

**Table 2:** Factors associated with incident TB during follow up [15 TB cases among 634 participants in 565 person-years of follow up]

Variable	Category	TB cases	Person-years of follow up	Incidence per 100 person years (95 % CI)	Univariable analysis		Multivariable analysis	
					Unadjusted HR [95 % CI]	*p*-value	Adjusted HR [95 % CI]	*p*-value
	Total population	15	565	2.7 (1.6–4.4)				
Gender	Female	13	458	2.8 (1.6–4.9)	1			
	Male	2	107	1.9 (0.5–7.4)	0.66 (0.15–2.91)	0.560		
Age	<35 years	6	258	2.3 (1.0–5.2)	1			
	≥35 years	9	307	2.9 (1.5–5.6)	1.27 (0.45–3)	0.649		
Employed	No	11	338	3.3 (1.8–5.9)	1			
	Yes	4	227	1.8 (0.7–4.7)	0.53 (0.17–1.68)	0.264		
Ever smoked	No	10	440	2.3 (1.2–4.2)	1			
	Yes	5	125	4.0 (1.7–9.6)	1.76 (0.60–5.14)	0.322		
Currently drink alcohol	No	8	341	2.3 (1.2–4.7)	1			
	Yes	7	224	3.1 (1.5–6.5)	1.32 (0.48–3.64)	0.593		
Duration since HIV test	≥ 2 years	6	193	3.1 (1.4–6.9)	1	0.905		
	<2 years	5	207	2.4 (1.0–5.8)	0.78 (0.24–2.57)			
	missing	4	164	2.4 (0.9–6.9)	0.79 (0.22–2.82)			
On ART	No	7	209	3.4 (1.6–7.0)	1		1	
	Yes	8	356	2.2 (1.1–4.5)	0.65 (0.24–1.81)	0.461	0.68 (0.18–1.82)	0.458
Current or prior INH use	No	12	446	2.7 (1.5–4.7)	1			
	Yes	3	119	2.5 (0.8–7.8)	0.95 (0.27–3.36)	0.930		
Current CPT use	No	12	464	2.5 (1.4–4.4)	1			
	Yes	3	81	3.7 (1.2–11.4)	1.48 (0.42–5.26)	0.557		
Previous TB treatment	No	10	416	2.4 (1.3–4.5)	1			
	Yes	5	149	3.4 (1.4–8.1)	1.40 (0.48–4.10)	0.545		
CD4 count	≥500 cells/μl	8	335	2.4 (1.2–4.8)	1			
	350–500 cells/μl	6	184	3.3 (1.5–7.3)	1.35 (0.47–3.89)	0.828		
	missing	1	47	2.1 (0.3–15.2)	0.86 (0.11–6.90)			
BMI category	≥18.5 kg/m^2^	12	521	2.3 (1.3–4.1)	1		1	0.036
	<18.5 kg/m^2^	3	32	9.4 (3.0–29.1)	3.93 (1.11–13.92)	0.034	3.87 (1.09–13.73)	
	missing	0	12	0	-	1		

### Factors associated with incident TB in follow up

Table 2 shows findings from univariable and multivariable analyses of factors associated with incident TB in follow up. In univariable analyses, having a low body mass index (BMI) [HR 3.93 (95 % CI 1.11–13.92), *p* = 0.034] was the only factor which was significantly associated with the risk of incident TB. In the multivariable model adjusting for the effect being on ART and low BMI, having a low BMI remained independently associated with the risk of incident TB [HR 3.87 (95 % CI 1.09–13.73). All variables in the model met the proportional hazards assumption.

In a sub-group analysis which excluded participants who reported TB symptoms at enrolment, there were nine incident TB cases in 482 pyrs of follow up. The TB incidence in this group was 2.1 per 100 pyrs (95 % CI 1.1–4.1 per 100 pyrs). The TB incidence seemed lower among participants on ART compared to when they were not on ART [1.5 per 100 pyrs (95 % CI 0.5- 3.7 per 100 pyrs) vs 3.4 per 100 pyrs (95 % CI 0.5–3.9 per 100 pyrs) although confidence intervals overlapped. In univariable analyses, being on ART remained associated with a statistically insignificant trend towards lower risk of TB compared to not being on ART [HR 0.43 (95 % CI 0.11–1.60), *p* = 0.207] while having a low BMI was associated with a statistically insignificant trend towards higher risk of TB [HR 2.87 (95 % CI 0.36–23.11), *p* = 0.322]. Because of a low number of outcomes, multivariable analyses were not conducted for this sub-group analysis.

## Discussion

In this secondary analysis, the overall incidence of TB among participants with CD4 counts > 350 cells/μl was high. Low BMI was independently associated with increased incidence of TB while being on ART was not associated with risk of incident TB in this population. Neither CD4 count level nor duration since HIV testing was associated with increased risk of incident TB.

Our finding of increased risk of TB among those with low BMI supports findings from previous studies. BMI has been shown to be a good predictor of both prevalent and incident TB among HIV positive individuals and among the general population [12–14]. It is also a predictor of mortality and poorer outcomes among HIV positive individuals diagnosed with TB [15, 16]. This finding suggests that BMI calculation may be included in TB screening and diagnostic algorithms in order to increase sensitivity of existing algorithms. More research is needed on how best to include BMI in TB screening algorithms.

Within HIV clinic settings, patients with high CD4 counts represent newly diagnosed individuals with higher CD4 counts and those who may have initiated ART at lower CD4 counts but have reconstituted their immune system to higher CD4 count levels. The TB incidence estimate from this analysis were comparable to the 2-3 cases per 100 person-years reported among similar cohorts but higher than the general population rate estimated to be 1000 cases per 100 000 population [13, 17–19]. ART is one of the most effective tools for preventing TB among individuals with HIV. However the effectiveness of ART as a tool for preventing TB is limited by the CD4 count levels and by the extent to which ART restores TB specific immune responses. Earlier initiation of ART prevents CD4 count decline and might preserve TB specific immune responses [6, 7]. The high rates of TB among those who had not initiated ART therefore support the need for earlier initiation of ART as a tool to prevent TB. Additional interventions such as TB screening, isoniazid preventive therapy and TB infection control are also needed to complement the ART. Not being on ART is a known risk factor for the development of opportunistic infections even at high CD4 counts, [9, 20, 21]. The individual and population-level impact of earlier ART initiation on TB incidence and outcomes is being investigated in trials of the universal test and treat [22, 23].

Half the incident TB cases occurred in the first 6 months of follow up suggesting they may have been prevalent or incubating cases at enrolment (although individuals diagnosed in the first 3 months were considered as prevalent TB). This finding highlights the continued need for intensified case finding at high CD4 counts regardless ART status [24]. Studies have suggested that the four symptom TB screening algorithm may be less sensitive and specific among individuals taking ART and receiving regular screening for TB [25, 26]. There is need to develop more sensitive and specific symptom screening algorithms that best identify individuals who require further evaluation for TB.

This analysis is based on a study which identified and enrolled participants with high CD4 counts in order to estimate TB incidence. We included a sub-group analysis excluding participants who reported symptoms at enrolment and likely excluded most prevalent TB at enrolment. However our analysis also had some limitations. Firstly the study had strict inclusion criteria for entry into the study. These criteria were necessary because the study was meant to be a TB vaccine preparedness study aimed at estimating incidence in populations that would be eligible for vaccine trials. The strict criteria may limit the extent to which findings are generalizable to other settings. Our study population was 80 % female likely as a result of the high CD4 count requirement which more women are likely to have from earlier diagnosis, linkage and retention in care [27–30]. Since rates were high in this population, we expect them to be higher in populations with lower CD4 counts. Secondly we had a limited sample size and duration of follow up. Because the incidence of TB decreases with increasing duration on ART, the shorter duration of follow up may have overestimated overall TB incidence in this cohort. Thirdly, because of budgetary constraints, there was sub-optimal follow up of participants in the study and most incident cases were diagnosed by the clinics. As a result we may have under ascertained the true number of TB cases in the study population. However both clinics had access to smear microscopy, culture and (at a later stage of the study) Xpert MTB/Rif and were able to refer individuals for chest radiographs and diagnosis of extra-pulmonary TB as part of routine TB and HIV care. This minimised the under-ascertainment of incident cases. The small number of outcomes meant very few variables could be included in multivariable cox proportional hazards regression models to adjust for confounding. Despite these limitations the study contributes relevant cohort data on the incidence of TB in an important population.

## Conclusion

The incidence of TB was high in this population of HIV positive individuals with relatively high CD4 counts. Intensified case finding needs to be strengthened regardless of CD4 count or ART status.

## Abbreviations

ART, antiretroviral therapy; BMI, body mass index; CD4, cluster of differentiation 4; CPT, cotrimoxazole preventive therapy; GCP, good clinical practice; HIV, human immunodeficiency virus; ICF, intensified case finding; IPT, isoniazid preventive therapy; TB, tuberculosis; WHO, World Health Organisation

## Additional files

Additional file 1: Enrolment form. (DOCX 35 kb) Additional file 2: Vaccine acceptance form. (DOCX 25 kb) Additional file 3: Baseline results form. (DOCX 23 kb) Additional file 4: Chest X-ray reading form. (DOCX 41 kb) Additional file 5: Study follow up form. (DOCX 33 kb) Additional file 6: Follow up results form. (DOCX 22 kb) Additional file 7: Enrolment and follow up database. (XLS 2101 kb) Additional file 8: TB cases database. (XLS 31 kb)
