# Supplementary material for: The incidence of tuberculosis among hiv-positive individuals with high CD4 counts: implications for policy
Source: BMC Infect Dis. 2016 Jun 10;16:266. doi: 10.1186/s12879-016-1598-8 (PMC4901468; doi:10.1186/s12879-016-1598-8)
Supplement: Additional file 1: — Enrolment form. (DOCX 35 kb) [file 12879_2016_1598_MOESM1_ESM.docx]

**MEASURING TB INCIDENCE IN EARLY HIV DISEASE**

**VISIT 1: ENROLMENT QUESTIONNAIRE**

| sex | **1** | **Sex**  01=Male 02=Female | \|___\|___\| |
| --- | --- | --- | --- |
| dob | **2** | 01/111/1119=if day, month and year not known  01/111/yyyy if day and month are not known  01/mmm/yyyy if day is not known | \|___\|___\|/\|___\|___\|___\|/\|___\|___\|___\|___\| |

**“I am going to ask you some questions your employment.”**

| employ | **3** | **How would you describe your employment situation at the moment?**  00= Unemployed looking for work  01=Unemployed not looking for work  02= Unable to work - Receive social grant  03=Student/pupil/learner  04=Self-employed – part time less than 40 hours per week  05=Self-employed – full time 40 hours or more per week  06=Employed, less than 40 hours per week  07=Employed, full time (40 hours or more)  08=Other  **If Other above** Write employment situation🡪 | \|___\|___\| |
| --- | --- | --- | --- |
| minvr | **4** | **Have you ever worked in the mines?**  00=No; 01=Yes | \|___\|___\| |
| minyrs | **5** | **If you have worked in the mines, for how many years did you work in the mines?**  97=N/A; 99=DK | \|___\|___\| |
| minug | **6** | **If you have worked in the mines, have you ever worked underground?**  00=No; 01=Yes; 07=N/A | \|___\|___\| |
| hcwevr | **7** | **Have you ever been a health care worker, i.e. worked in a hospital or clinic setting AND been close enough to talk to patients / been close enough to be coughed on by patients / worked in medical laboratory?** 00=No; 01=Yes | \|___\|___\| |
| hcwcur | **8** | **Are you currently a health care worker i.e. working in a hospital or clinic setting AND close enough to talk to patients / close enough to be coughed on by patients / working in medical laboratory?** 00=No; 01=Yes | \|___\|___\| |
| army | **9** | **Have you ever been in the military/ army?**  00=No; 01=Yes | \|___\|___\| |
| prison | **10** | **Have you ever been in prison/jail or worked in a prison/jail?**  00=No; 01=Yes | \|___\|___\| |
| hosp | **11** | **Have you stayed in a hospital for a period longer than 1 night in the past year?**  00=No; 01=Yes | \|___\|___\| |

**“I am going to ask you some questions about cigarette smoking and alcohol consumption. People have different habits and there is no right or wrong answer.”**

| smever | **12** | **Have you smoked at least 100 cigarettes in your entire life?**  00=No; 01=Yes; 99=Don’t know | \|___\|___\| |
| --- | --- | --- | --- |
| smcur | **13** | **Do you now smoke cigarettes every day, some days, or not at all?**  00=Not at all; 01=Every day; 02=Some days | \|___\|___\| |
| smnum | **14** | **Currently, how many cigarettes per day do you smoke on average?**  97=N/A; 99=D/K | \|___\|___\| |
| smstrt | **15** | **In what year did you start smoking?**  1117=N/A; 1119=D/K | \|___\|___\|___\|___\| |
| smstop | **16** | **In what year did you stop smoking?**  1117=N/A; 1119=D/K | \|___\|___\|___\|___\| |
| alcevr | **17** | **Have you ever drunk any kind of alcohol?**  01=Yes; 02=No; 98=missing CRF data; 99=D/K | \|___\|___\| |
| alcunit | **18** | **How many drinks do/did you consume *on average per week*?**   \|  \| \| **Weekly** \| **Monthly** \| \| --- \| --- \| --- \| --- \| \| 1 can beer/cider \| 1 unit \|  \|  \| \| 1 pint beer/cider \| 2 units \|  \|  \| \| 750ml bottle beer/cider \| 3 units \|  \|  \| \| 1small (120ml) glass wine \| 1 unit \|  \|  \| \| 250ml glass wine \| 2 units \|  \|  \| \| 1 tot whisky / brandy \| 1 unit \|  \|  \| \| 1 nip brandy (200ml) \| 8 units \|  \|  \| \| 1 half-jack brandy (375ml) \| 16 units \|  \|  \| \| 1 sakiya (carton) beer \| 4 units \|  \|  \| | Ask about all the different types and quantities of drinks that the participant consumes and then add up and enter the total number of units.  000=NONE  998=missing CRF data; 999=D/K  \|___\|___\|___\| units per week |

**“I am going to ask you about other illnesses you may have had in the past”**

| smever | **19** | **Have you ever been diagnosed of diabetes?**  00=No; 01=Yes; 09=Don’t know | \|___\|___\| |
| --- | --- | --- | --- |
| smcur | **20** | **If yes in what years was the diagnosis made?**  1117=N/A; 1119=D/K | \|___\|___\|___\|___\| |
| DMtyp | **21** | **If you have Diabetes, do you know what type of Diabetes you have?**  01=Type 1 DM (IDDM); 02=Type 2 DM (NIDDM); 97=N/A (don’t have DM); 98=missing CRF data; 99=D/K | \|___\|___\| |
| DMtr | **22** | **If you have Diabetes, what treatment are you on?**  01=Insulin; 02=Tablets; 03=Diet; 97=N/A (don’t have DM); 98=missing CRF data; 99=D/K | \|___\|___\| |
| livdis | **23** | **Have you ever been diagnosed of liver disease in the past**  00=No; 01=Yes; 09=Don’t know | \|___\|___\| |
| livjau | **24** | **Have you had yellow eyes in the past**  00=No; 01=Yes; 99=Don’t know | \|___\|___\| |

**“I am going to ask you about your symptoms.”**

|  |  | **Symptom** | **Is symptom present?**  00=No; 01=Yes; 99=D/K | **Duration of symptom**  *For how long have you had symptom?*  997=N/A; 999=D/K |
| --- | --- | --- | --- | --- |
| cou0  coudu0 | **26** | **Do you have a cough?** | \|___\|___\| | \|___\|___\|___\| days |
| pro0  produ0 | **26** | **Are you currently coughing up sputum (spit from the chest)?** | \|___\|___\| | \|___\|___\|___\| days |
| ns0  nsdu0 | **27** | **Do you sweat so much at night that your clothes or pillows are soaking wet?** | \|___\|___\| | \|___\|___\|___\| days |
| uwl0 | **28** | **Have you lost weight (without trying) so that your clothes have become looser?** | \|___\|___\| | N/A |
| fev0  fevdu0 | **29** | **Have you had any fevers (high temperature)?** | \|___\|___\| | \|___\|___\|___\| days |
| hem0  hemdu0 | **30** | **Have you coughed up any blood?** | \|___\|___\| | \|___\|___\|___\| days |
| sob0  sobdu0 | **31** | **Have you experienced any difficulty breathing in the last 1 month?** | \|___\|___\| | \|___\|___\|___\| days |
| cp0  cpdu0 | **32** | **Have you experienced any chest pain in the last 1 month?** | \|___\|___\| | \|___\|___\|___\| days |
| loa0  loadu0 | **33** | **Have you experienced any loss of appetite (not hungry / not wanting to eat) in the last 1 month?** | \|___\|___\| | \|___\|___\|___\| days |
| fat0  fatdu0 | **34** | **Do you feel tired most of the time?** | \|___\|___\| | \|___\|___\|___\| days |
| osxs01 | **35** | **Do you have any other symptoms?**  **If yes, specify:** | \|___\|___\| | \|___\|___\|___\| days |
| osxs02 | **36** | **Do you have any other symptoms?**  **If yes, specify:** | \|___\|___\| | \|___\|___\|___\| days |
| osxs03 | **37** | **Do you have any other symptoms?**  **If yes, specify:** | \|___\|___\| | \|___\|___\|___\| days |
| pregenrol | **38** | **Are you pregnant**  00=No; 01=Yes;07=N/A ; 09=Don’t know | \|___\|___\| |  |
| bslnsxs | **39** | **Has the study participant had any symptoms suggestive of TB (i.e. any symptoms 26-30?**  00=No; 01=Yes; | \|___\|___\| | bslnsxs |
|  |  | **If yes give sputum cup and instructions for sputum collection. Enter specimen number/ bar code here**  **Also give a CXR request form** |  |  |
|  |  | **If female give cup and obtain urine specimen to exclude pregnancy** |  |  |

**“I will now talk to you about tuberculosis.”**

| conevr | **40** | **Have you EVER been in contact with anyone with TB?**  *(close enough to talk to someone with TB) 0* 0=No; 01=Yes; 99=Don’t know | \|___\|___\| | |
| --- | --- | --- | --- | --- |
| condt | **41** | **When was the last time you were in contact with anyone with TB?***(close enough to talk to someone with TB)*  *ENTER YEAR* 1117=N/A; 1119=D/K | \|___\|___\|___\|___\| | |
| con1yr | **42** | **Have you been in contact with anyone with TB in the past 1 year?**  *(close enough to talk to someone with TB) 0* 0=No; 01=Yes; 99=Don’t know | \|___\|___\| | |
| TBlvev | **43** | **Have you EVER lived with anyone with TB?**  00=No; 01=Yes; 99=Don’t know | | \|___\|___\| |
| TBlvdt | **44** | **When was the last time you lived with anyone with TB?**  *ENTER YEAR* 1117=N/A; 1119=D/K | | \|___\|___\|___\|___\| |
| TBlv1y | **45** | **Have you lived with anyone with TB in the past 1 year?**  00=No; 01=Yes; 99=Don’t know | | \|___\|___\| |
| prTBtx | **46** | **Have you ever been treated for TB previously?**  00=No; 01=Yes; 99=Don’t know | | \|___\|___\| |
| prTB | **47** | **Number of previous TB episodes**  7=N/A; 9=D/K | | \|___\|___\| |
| prTBdt | **48** | **Year treatment was started for most recent previous episode of TB**  1117=N/A 1119=Don’t know | | \|___\|___\|___\|___\| |
| TBtx | **49** | **Are you currently being treated for TB?**  00=No; 01=Yes; 99=Don’t know | | \|___\|___\| |
|  | **50** | **If yes, when was the date you started TB treatment?** | | \|___\|___\|/\|___\|___\|___\|/\|___\|___\|___\|___\| |
|  |  | **If yes please obtain the name of the clinic where patient is registered in order to verify information** | |  |
| TBtx3m | **51** | **Have you been treated for TB in the last 3 months?**  00=No; 01=Yes; 99=Don’t know | | \|___\|___\| |
| inhevr | **52** | **Have you ever received isoniazid (INH) preventive therapy?**  00=No; 01=Yes; 99=Don’t know | | \|___\|___\| |
| inhnow | **53** | **Are you ever currently taking isoniazid (INH) preventive therapy?**  00=No; 01=Yes; 99=Don’t know | | \|___\|___\| |
| inhdt | **54** | **Year of starting isoniazid (INH).(yyyy)**  1117=N/A 1119=Don’t know | | \|___\|___\|___\|___\| |

**“I will now ask about HIV and medications taken for HIV.”**

| hivtstdte | **55** | **When was the date of your first HIV test?** | \|___\|___\|/\|___\|___\|___\|/\|___\|___\|___\|___\| |
| --- | --- | --- | --- |
| ctx | **56** | **Are you taking co-trimoxazole (Bactrim) or dapsone?**  00=No; 01=Yes; 99=Don’t know | \|___\|___\| |
| artevr | **57** | **Have you ever received antiretroviral therapy (i.e. treatment for HIV)??**  00=No; 01=Yes; 99=Don’t know | \|___\|___\| |
| artnow | **58** | **Are you currently taking antiretrovirals (i.e treatment for HIV)?**  00=No; 01=Yes; 99=Don’t know | \|___\|___\| |
|  |  | **If yes, please ensure details of clinic where ART service is provided are included in the locator form** |  |
| cd4 | **59** | **Have you had a CD4 count test done in the last 3 months?** 00=No; 01=Yes; 99=Don’t know | \|___\|___\| |
| cd4dte | **60** | **What was the date of your last CD4 count** | \|___\|___\|/\|___\|___\|___\|/\|___\|___\|___\|___\| |
| cd4vle | **61** | **What was the value of your CD4 count?** | \|___\|___\|___\|___\| |

**“I will now weigh and measure your height.”**

| wt0 | **62** | **Weight today in kilograms:** | \|___\|___\|\|___\|**.**\|___\| |
| --- | --- | --- | --- |
| ht0 | **63** | **Height today in centimeters:** | \|___\|___\|\|___\|**.**\|___\| |

**“I will now give you a date on which you need to return for a follow up visit”.**

| dtevis | **64** | **Date of follow up visit** | \|___\|___\|/\|___\|___\|___\|/\|___\|___\|___\|___\| |
| --- | --- | --- | --- |

Completed by |___||___| Verified by |___||___| First entry: |___||___| Double entry|___||___|
