# Supplementary material for: The incidence of tuberculosis among hiv-positive individuals with high CD4 counts: implications for policy
Source: BMC Infect Dis. 2016 Jun 10;16:266. doi: 10.1186/s12879-016-1598-8 (PMC4901468; doi:10.1186/s12879-016-1598-8)
Supplement: Additional file 2: — Vaccine acceptance form. (DOCX 25 kb) [file 12879_2016_1598_MOESM2_ESM.docx]

**MEASURING TB INCIDENCE IN EARLY HIV DISEASE**

**TB VACCINE ACCEPTABILITY FORM**

***Instructions to Research Nurse:***

Part of this study involves finding from participants if they would be willing to get a new TB vaccine should it become available. This form intends to find out from participant if they would be willing to receive a TB vaccine should one become available and why. Although this form is structured please allow the participant to answer the question as strongly agree, agree, disagree, strongly disagree or don’t know but ask them to explain their answer and give reasons for their answers in the space provided.

| sex | **1** | **Sex**  01=Male 02=Female | \|___\|___\| |
| --- | --- | --- | --- |
| dob | **2** | **What is your date of birth? (dd/mmm/yyyy)**  11/111/1119=if day, month and year not known  11/1111/yyyy if day and month are not known  11/mmm/yyyy if day is not known | \|___\|___\|/\|___\|___\|___\|/\|___\|___\|___\|___\| |

**Vaccine Acceptance**

| tbvaccex | **1** | **I would be willing to take part in studies to test if a new injection to prevent TB works or not**  01= Strongly agree 02=Agree 03=Disagree 04= Strongly disagree 99=Don’t know  **Give a reason for your answer** | \|___\|___\| |
| --- | --- | --- | --- |
| tbvaccexfam | **2** | **I would be willing to let my family/household members take part in studies to test if a new injection to prevent TB works or not**  01= Strongly agree 02=Agree 03=Disagree 04= Strongly disagree 99=Don’t know  **Who in your family or household would you be willing to get the injection for and why?** | \|___\|___\| |
| tbvaccexchil | **3** | **I would be willing to let my children take part in studies to test if a new injection to prevent TB works or not**  01= Strongly agree 02=Agree 03=Disagree 04= Strongly disagree 99=Don’t know  **Give a reason for your answer** | \|___\|___\| |
| tbinjwork | **4** | **I would be willing to let my children take part in studies to test a new injection to prevent TB if I was convinced it would work**  01= Strongly agree 02=Agree 03=Disagree 04= Strongly disagree 99=Don’t know  **Give a reason for your answer** | \|___\|___\| |
| tbinjpain | **5** | **I would be willing to let my children take part in studies to test a new injection to prevent TB even if it was painful**  01= Strongly agree 02=Agree 03=Disagree 04= Strongly disagree 99=Don’t know  **Give a reason for your answer** | \|___\|___\| |
| tbinjsick | **6** | **I would be willing to let my children take part in studies to test a new injection to prevent TB even if it made me a little sick (like vomiting, rashes, fever)**  01= Strongly agree 02=Agree 03=Disagree 04= Strongly disagree 99=Don’t know  **Give a reason for your answer** | \|___\|___\| |
| tbinjavail | **7** | **Once a new injection to prevent TB has been shown to work in studies, I would be willing to get it if it became available at my clinic**  01= Strongly agree 02=Agree 03=Disagree 04= Strongly disagree 99=Don’t know  **Give a reason for your answer** | \|___\|___\| |
| tbinjavailfam | **8** | **Once a new injection to prevent TB has been shown to work in studies, I would be willing to get it for my family or household members if it became available at my clinic**  01= Strongly agree 02=Agree 03=Disagree 04= Strongly disagree 99=Don’t know  **Who in your family or household would you be willing to get the vaccine for and why?** |  |
| tbinjchil | **9** | **Once the new injection to prevent TB has been shown to work in studies, I would be willing to get it for my children if it became available at my clinic**  01= Strongly agree 02=Agree 03=Disagree 04= Strongly disagree 99=Don’t know  **Give a reason for your answer** | \|___\|___\| |
| tbinjsick | **10** | **Once the new injection to prevent TB has been shown to work in studies, I would be willing to get it even if it made me a little sick (like vomiting, rashes, fever)**  01= Strongly agree 02=Agree 03=Disagree 04= Strongly disagree 99=Don’t know  **Give a reason for your answer** | \|___\|___\| |
| tbinjpay | **11** | **Once the new injection to prevent TB has been shown to work in studies I would be willing to get it even if I had to pay for it**  01= Strongly agree 02=Agree 03=Disagree 04= Strongly disagree 99=Don’t know  **Give a reason for your answer** | \|___\|___\| |

**TB knowledge**

| tbrisk | **12** | **I consider myself at risk of getting TB**  01= Strongly agree 02=Agree 03=Disagree 04= Strongly disagree 99=Don’t know | \|___\|___\| |
| --- | --- | --- | --- |
| tblving | **13** | **TB can be acquired by living in the same house with someone with TB**  01= Strongly agree 02=Agree 03=Disagree 04= Strongly disagree 99=Don’t know | \|___\|___\| |
| tbspace | **14** | **TB can be passed to another through sharing a closed space with someone who has TB**  01= Strongly agree 02=Agree 03=Disagree 04= Strongly disagree 99=Don’t know | \|___\|___\| |
| tbplts | **15** | **TB can be passed to another through sharing plates or clothes**  01= Strongly agree 02=Agree 03=Disagree 04= Strongly disagree 99=Don’t know | \|___\|___\| |
| tbwitch | **16** | **TB can be a result of witchcraft**  01= Strongly agree 02=Agree 03=Disagree 04= Strongly disagree 99=Don’t know | \|___\|___\| |
| tbhiv | **17** | **Being HIV positive increases someone’s chances of getting TB**  01= Strongly agree 02=Agree 03=Disagree 04= Strongly disagree 99=Don’t know | \|___\|___\| |
| tbcough | **18** | **Anyone who is coughing for more than two weeks could have TB**  01= Strongly agree 02=Agree 03=Disagree 04= Strongly disagree 99=Don’t know | \|___\|___\| |
| tbsweats | **19** | **Anyone who has night sweats for more than two weeks could have TB**  01= Strongly agree 02=Agree 03=Disagree 04= Strongly disagree 99=Don’t know | \|___\|___\| |
| tbfever | **20** | **Anyone who has fever for more than two weeks may have TB**  01= Strongly agree 02=Agree 03=Disagree 04= Strongly disagree 99=Don’t know | \|___\|___\| |
| tbweight | **21** | **Anyone who is losing weight may have TB**  01= Strongly agree 02=Agree 03=Disagree 04= Strongly disagree 99=Don’t know | \|___\| |
| hosp | **22** | **I have been treated for TB in the past**  01= Strongly agree 02=Agree 03=Disagree 04= Strongly disagree 99=Don’t know | \|___\|___\| |

Completed by |___||___| Verified by |___||___| First entry: |___||___| Double entry|___||___|
