# Supplementary material for: The incidence of tuberculosis among hiv-positive individuals with high CD4 counts: implications for policy
Source: BMC Infect Dis. 2016 Jun 10;16:266. doi: 10.1186/s12879-016-1598-8 (PMC4901468; doi:10.1186/s12879-016-1598-8)
Supplement: Additional file 3: — Baseline results form. (DOCX 23 kb) [file 12879_2016_1598_MOESM3_ESM.docx]

**MEASURING TB INCIDENCE IN EARLY HIV DISEASE**

**BASELINE SCREENING AND ENROLMENT RESULTS FORM**

1. **CD4 results (abstract from laboratory printout of results)**

| cd4  cd4dt | **1.** | **CD4 count done**  01=yes 02=no  \|___\|___\| | **CD4+ T-cell count result**  (0-3000) | **CD4 count (10^6 cells per liter)**  9997=N/A (not done) 9999=DK | **Specimen date (dd/mmm/yyyy)**  **11/111/1117=N/A; 11/111/1119=D/K** |
| --- | --- | --- | --- | --- | --- |
|  |  |  |  | \|___\|___\|___\|___\| | \|___\|___\|/\|___\|___\|___\|/\|___\|___\|___\|___\| |

1. **CXR results (abstract results from CXR report)**

| dcxr | **2** | **Date CXR taken**  **11/111/1117=N/A; 11/111/1119=D/K** | \|___\|___\|/\|___\|___\|___\|/\|___\|___\|___\|___\| |
| --- | --- | --- | --- |
| dcxrr | **3** | **Date CXR read**  **11/111/1117=N/A; 11/111/1119=D/K** | \|___\|___\|/\|___\|___\|___\|/\|___\|___\|___\|___\| |
| cxrqual | **4** | **Is CXR of acceptable quality**  01=yes 02=no | \|___\|___\| |
| norcxr | **5** | **Is the CXR normal**  01=yes 02=no 99=N/A | \|___\|___\| |
| cxrabs | **6** | **If CXR is abnormal, are abnormalities consistent with TB disease?**  01=yes 02=no 99=N/A | \|___\|___\| |
| specTBab | **7** | **If yes specify** |  |
| acttb | **8** | **Is there evidence of active TB?** 00=no active TB,  01=definite active TB,  02=probable active TB,  03=possible active TB | \|___\|___\| |
| oldtb | **9** | **Is there evidence of previous TB**  00=no previous,  01=definite previous TB,  02=probable previous TB,  03=possible previous TB | \|___\|___\| |
| silic | **10** | **Is there evidence of silicosis**  00=none  01= definite  02=possible | \|___\|___\| |
| other | **11** | **Are there other abnormalities not consistent with TB**  00=none 01=yes | \|___\|___\| |
| spec | **12** | **If yes specify** | \| |

1. **Sputum smear results (abstract results from laboratory printout of results)**

| spcdt | **11** | Date specimen collected  11/111/1117=N/A ; 11/111/1119=DK | \|___\|___\|/\|___\|___\|___\|/\|___\|___\|___\|___\| |
| --- | --- | --- | --- |
| spclab | **12** | Date specimen received by the lab  11/111/1117=N/A ; 11/111/1119=DK | \|___\|___\|/\|___\|___\|___\|/\|___\|___\|___\|___\| |
| ssm | **13** | **Sputum AFB results and date:**   \| 00=AFB Negative \| \| --- \| \| 01=AFB Positive 1+ \| \| 02=AFB Positive 2+ \| \| 03=AFB Positive 3+ \| \| 04=AFB Positive scanty \| \| 05=Non productive (inadequate specimen) \| \| 6=Other: \| \| 07=Not applicable (i.e. not done) \| \| 09=Don’t know \| | **AFB result** |

1. **Sputum culture results (abstract results from laboratory printout of results)**

| spcdt | **14** | **Date specimen collected**  11/111/1117=N/A ; 11/111/1119=DK | \|___\|___\|/\|___\|___\|___\|/\|___\|___\|___\|___\| |
| --- | --- | --- | --- |
| spclab | **15** | **Date specimen received by the lab**  11/111/1117=N/A ; 11/111/1119=DK | \|___\|___\|/\|___\|___\|___\|/\|___\|___\|___\|___\| |
| cx+dt | **16** | **Date specimen turned culture positive**  11/111/1117=N/A ; 11/111/1119=DK | \|___\|___\|/\|___\|___\|___\|/\|___\|___\|___\|___\| |
| spcx  spcxdt | **17** | **Mycobacterial culture results**   \| 00=Negative (no growth) \| \| --- \| \| 01=Positive MTB only \| \| 02=Positive MTB & other organism  (record other organism below) \| \| 03=Positive other organism only  (record other organism below) \| \| 04=Contaminated \| \| 05=Inadequate specimen \| \| 07=Not applicable (i.e. not requested) \| \| 08=Other: \| \| 09=Don’t know \| | \|___\|___\| |

Completed by |___||___| Verified by |___||___| First entry: |___||___| Double entry|___||___|
