# Supplementary material for: The incidence of tuberculosis among hiv-positive individuals with high CD4 counts: implications for policy
Source: BMC Infect Dis. 2016 Jun 10;16:266. doi: 10.1186/s12879-016-1598-8 (PMC4901468; doi:10.1186/s12879-016-1598-8)
Supplement: Additional file 4: — Chest X-ray reading form. (DOCX 41 kb) [file 12879_2016_1598_MOESM4_ESM.docx]

**MEASURING TB INCIDENCE IN EARLY HIV DISEASE**

**Chest Radiograph Case Report** Form (to be completed by Radiologist)

| CXRdt | **1** | **Date of chest x-ray** *(if missing enter 11/111/1117 and score out remainder of section):* \|___\|___\|/\|___\|___\|___\|/\|___\|___\|___\|___\| | | | |
| --- | --- | --- | --- | --- | --- |
| QUAL | **2** | **X-ray quality**  (IF QUALITY IS NOT ACCEPTABLE SCORE OUT REST OF THIS SECTION)  Not acceptable = *Over-exposed/Under-exposed; Processor problem, e.g. fogging or staining;*  *Movement artefact; Poor positioning* | \|___\|  0=Not acceptable; 1=Acceptable | | |
| XRnor | **3** | **Is CXR NORMAL?**  0=NO; 1=YES  ***** IF CXR IS NORMAL, STOP HERE. ***** | \|___\| | | |
| LBLRPO | **4** | **Lobular parenchymal opacity** |  | **Right** | **Left** |
|  |  |  | Upper zone | 🞎 | 🞎 |
|  |  |  | Mid Zone | 🞎 | 🞎 |
|  |  |  | Lower zone | 🞎 | 🞎 |
|  |  |  | 0=Absent;1=Present | | |
| SSPO | **5** | **Sub-segmental parenchymal opacity** |  | **Right** | **Left** |
|  |  |  | Upper zone | 🞎 | 🞎 |
|  |  |  | Mid Zone | 🞎 | 🞎 |
|  |  |  | Lower zone | 🞎 | 🞎 |
|  |  |  | 0=Absent;1=Present | | |
| LBRPO | **6** | **Lobar parenchymal opacity** |  | **Right** | **Left** |
|  |  |  | Upper zone | 🞎 | 🞎 |
|  |  |  | Mid Zone | 🞎 | 🞎 |
|  |  |  | Lower zone | 🞎 | 🞎 |
|  |  |  | 0=Absent;1=Present | | |
| CAV | **7** | **Cavitation** |  | **Right** | **Left** |
|  |  |  | Upper zone | 🞎 | 🞎 |
|  |  |  | Mid Zone | 🞎 | 🞎 |
|  |  |  | Lower zone | 🞎 | 🞎 |
|  |  |  | 0=Absent;1=Present | | |

| PCHD | **8** | **Patchy disease** |  | **Right** | **Left** |
| --- | --- | --- | --- | --- | --- |
|  |  |  | Upper zone | 🞎 | 🞎 |
|  |  |  | Mid Zone | 🞎 | 🞎 |
|  |  |  | Lower zone | 🞎 | 🞎 |
|  |  |  | 0=Absent;1=Present | | |
| SEGD | **9** | **Segmental disease** |  | **Right** | **Left** |
|  |  |  | Upper zone | 🞎 | 🞎 |
|  |  |  | Mid Zone | 🞎 | 🞎 |
|  |  |  | Lower zone | 🞎 | 🞎 |
|  |  |  | 0=Absent;1=Present | | |
| BRALD | **10** | **Broncho-alveolar disease** |  | **Right** | **Left** |
|  |  |  | Upper zone | 🞎 | 🞎 |
|  |  |  | Mid Zone | 🞎 | 🞎 |
|  |  |  | Lower zone | 🞎 | 🞎 |
|  |  |  | 0=Absent;1=Present | | |
| MIL | **11** | **Miliary disease** |  | **Right** | **Left** |
|  |  |  | Upper zone | 🞎 | 🞎 |
|  |  |  | Mid Zone | 🞎 | 🞎 |
|  |  |  | Lower zone | 🞎 | 🞎 |
|  |  |  | 0=Absent;1=Present | | |
| NOD | **12** | **Nodules** |  | **Right** | **Left** |
|  |  |  | Upper zone | 🞎 | 🞎 |
|  |  |  | Mid Zone | 🞎 | 🞎 |
|  |  |  | Lower zone | 🞎 | 🞎 |
|  |  |  | 0=Absent;1=Present | | |
| CIC | **13** | **Cicatrisation** |  | **Right** | **Left** |
|  |  |  | Upper zone | 🞎 | 🞎 |
|  |  |  | Mid Zone | 🞎 | 🞎 |
|  |  |  | Lower zone | 🞎 | 🞎 |
|  |  |  | 0=Absent;1=Present | | |
| FIBBR | **14** | **Fibrotic bronchiectasis** |  | **Right** | **Left** |
|  |  |  | Upper zone | 🞎 | 🞎 |
|  |  |  | Mid Zone | 🞎 | 🞎 |
|  |  |  | Lower zone | 🞎 | 🞎 |
|  |  |  | 0=Absent;1=Present | | |
| CICB | **15** | **Cicatrisation bullae** |  | **Right** | **Left** |
|  |  |  | Upper zone | 🞎 | 🞎 |
|  |  |  | Mid Zone | 🞎 | 🞎 |
|  |  |  | Lower zone | 🞎 | 🞎 |
|  |  |  | 0=Absent;1=Present | | |
| CSO | **16** | **Costophrenic sulcus obliteration** |  | **Right** | **Left** |
|  |  |  | Upper zone | 🞎 | 🞎 |
|  |  |  | Mid Zone | 🞎 | 🞎 |
|  |  |  | Lower zone | 🞎 | 🞎 |
|  |  |  | 0=Absent;1=Present | | |
| FVL | **17** | **Fibrotic volume loss** |  | **Right** | **Left** |
|  |  |  | Upper zone | 🞎 | 🞎 |
|  |  |  | Mid Zone | 🞎 | 🞎 |
|  |  |  | Lower zone | 🞎 | 🞎 |
|  |  |  | 0=Absent;1=Present | | |
| FIBC | **18** | **Fibrotic cavity** |  | **Right** | **Left** |
|  |  |  | Upper zone | 🞎 | 🞎 |
|  |  |  | Mid Zone | 🞎 | 🞎 |
|  |  |  | Lower zone | 🞎 | 🞎 |
|  |  |  | 0=Absent;1=Present | | |
| FIBNOD | **19** | **Fibronodular** |  | **Right** | **Left** |
|  |  |  | Upper zone | 🞎 | 🞎 |
|  |  |  | Mid Zone | 🞎 | 🞎 |
|  |  |  | Lower zone | 🞎 | 🞎 |
|  |  |  | 0=Absent;1=Present | | |

| HILDIS | **20** | **Hilar distortion** |  | **Right** | | | **Left** |
| --- | --- | --- | --- | --- | --- | --- | --- |
|  |  |  | Upper zone | 🞎 | | | 🞎 |
|  |  |  | Mid Zone | 🞎 | | | 🞎 |
|  |  |  | Lower zone | 🞎 | | | 🞎 |
|  |  |  | 0=Absent;1=Present | | | | |
| TRADEV | **21** | **Tracheal deviation**  0=Absent;1=Present | **R**: \|___\| | | **L**: \|___\| | | |
| CALC | **22** | **Calcifications** |  | **Right** | | | **Left** |
|  |  |  | Upper zone | 🞎 | | | 🞎 |
|  |  |  | Mid Zone | 🞎 | | | 🞎 |
|  |  |  | Lower zone | 🞎 | | | 🞎 |
|  |  |  | 0=Absent;1=Present | | | | |
| OTH | **23** | **Other**   \| Specify: \| \| --- \| |  | **Right** | | | **Left** |
|  |  |  | Upper zone | 🞎 | | | 🞎 |
|  |  |  | Mid Zone | 🞎 | | | 🞎 |
|  |  |  | Lower zone | 🞎 | | | 🞎 |
|  |  |  | 0=Absent;1=Present | | | | |
| LAD | **24** | **Lymphadenopathy** |  | **Right** | | | **Left** |
|  |  |  | Hilar | 🞎 | | | 🞎 |
|  |  |  | Mediastinal | 🞎 | | | 🞎 |
|  |  |  | Calcified | 🞎 | | | 🞎 |
|  |  |  | 0=Absent;1=Present | | | | |
| PLEFF | **25** | **Pleural effusion**  0=Absent; 1=Calcified; 2=Not calcified | **R**: \|___\| | | | **L**: \|___\| | |
| PLTHI | **26** | **Pleural thickening**  0=Absent;1=Present | **R**: \|___\| | | | **L**: \|___\| | |
| PEREF | **27** | **Pericardial effusion**  0=Absent; 1=Calcified; 2=Not calcified | \|___\| | | | | |
| DESTL | **28** | **Destroyed lung** |  | **Right** | | | **Left** |
|  |  |  | Upper zone | 🞎 | | | 🞎 |
|  |  |  | Mid Zone | 🞎 | | | 🞎 |
|  |  |  | Lower zone | 🞎 | | | 🞎 |
|  |  |  | 0=No;1=Yes | | | | |
| OTHTB | **29** | **Any other abnormality consistent with TB**  0=Absent;1=Present   \| If present, specify: \| \| --- \| | \|___\| | | | | |
| LNA | **30** | **Linear nodular arraignment**  0=Absent;1=Present | **R**: \|___\| | | **L**: \|___\| | | |
| HILF | **31** | **Hilar flare**  0=Absent;1=Present | **R**: \|___\| | | **L**: \|___\| | | |
| EXPRO | **32** | **Excessive Profusion**  0=Absent;1=Present | **R**: \|___\| | | **L**: \|___\| | | |
| SILIC | **33** | **Silicosis score**  none [0/0]; possible [0/1, 1/0]; definite [1/1, 2/2, 3/3] | \|___\|/\|___\| | | | | |
| ZONES | **34** | **Zone scores (0, 1, 2, 3, 4, 5, 6)**   - Number of lung regions involved by disease (infiltrate, cavity or effusion). - Each lung is divided into 3 zones by dividing the distance between the apex of the lung and the ipsilateral diaphragm (measured with a ruler) by 3. - The zone score represents the number of zones where visible disease is present. | \|___\| | | | | |
| SPLEN | **35** | **Splenomegaly**  0=Absent;1=Present | \|___\| | | | | |
| XRaTB | **36** | **Is there evidence of active TB?**   - 0=no active TB - 1=definite active TB - 2=probable active TB - 3=possible active TB | \|___\| | | | | |
| XRpTB | **37** | **Is there evidence of previous TB?**   - 0=no previous TB - 1=definite previous TB - 2=probable previous TB - 3=possible previous TB | \|___\| | | | | |
| TBGRAD | **38** | **TB Grading**   - 0= minimal (sum of lesions < 1/3 of lung) - 1= moderately advanced (sum of both sides < one lung field, cavity < 4 cms) - 2= far advanced (more severe than moderately advanced disease) - 9=N/A | \|___\| | | | | |
| XRoth | **39** | **If CXR is abnormal are there other abnormalities unrelated to TB or silicosis?**   - 00=no other abnormalities - 01=abnormal raised right hemidiaphragm - 02=lobar pneumonia - 03=solid mass - 04=abscess - 05=pneumothorax - 06=bullae - 07=trauma - 08=COPD - 09=congenital abnormality - 10=other - 99=N/A   ***PRIORITISE a, b, c*** | a. \|___\|___\| *If other record:*  b. \|___\|___\|  c. \|___\|___\| | | | | |

Completed by |___||___| Verified by |___||___| First entry: |___||___| Double entry|___||___|
