# Supplementary material for: The incidence of tuberculosis among hiv-positive individuals with high CD4 counts: implications for policy
Source: BMC Infect Dis. 2016 Jun 10;16:266. doi: 10.1186/s12879-016-1598-8 (PMC4901468; doi:10.1186/s12879-016-1598-8)
Supplement: Additional file 5: — Study follow up form. (DOCX 33 kb) [file 12879_2016_1598_MOESM5_ESM.docx]

**MEASURING TB INCIDENCE IN EARLY HIV DISEASE**

**FOLLOW UP FORM**

**FOLLOW UP VISIT 6 months 12 months**

| sex | **1** | **Sex**  01=Male 02=Female | \|___\|___\| |
| --- | --- | --- | --- |
| dob | **2** | **What is your date of birth? (dd/mmm/yyyy)**  01/111/1119=if day, month and year not known  01/1111/yyyy if day and month are not known  01/mmm/yyyy if day is not known | \|___\|___\|/\|___\|___\|___\|/\|___\|___\|___\|___\| |

**“I am going to ask you about your symptoms.”**

|  |  | **Symptom** | **Is symptom present?**  00=No; 01=Yes; 99=D/K | **Duration of symptom**  *For how long have you had symptom?*  997=N/A; 999=D/K |
| --- | --- | --- | --- | --- |
| cou6  coudu6 | **3** | **Do you have a cough?** | \|___\|___\| | \|___\|___\|___\| days |
| pro6  produ6 | **4** | **Are you currently coughing up sputum (spit from the chest)?** | \|___\|___\| | \|___\|___\|___\| days |
| ns6  nsdu6 | **5** | **Do you sweat so much at night that your clothes or pillows are soaking wet?** | \|___\|___\| | \|___\|___\|___\| days |
| uwl6 | **6** | **Have you lost any weight (without trying) so that your clothes have become looser?** | \|___\|___\| | \|___\|___\|___\| days |
| fev6  fevdu6 | **7** | **Have you had any fevers (high temperature)?** | \|___\|___\| | \|___\|___\|___\| days |
| hem6  hemdu6 | **8** | **Have you coughed up any blood in the last 1 month?** | \|___\|___\| | \|___\|___\|___\| days |
| sob6  sobdu6 | **9** | **Have you experienced any difficulty breathing in the last 1 month?** | \|___\|___\| | \|___\|___\|___\| days |
| cp6  cpdu6 | **10** | **Have you experienced any chest pain in the last 1 month?** | \|___\|___\| | \|___\|___\|___\| days |
| loa6  loadu6 | **11** | **Have you experienced any loss of appetite (not hungry / not wanting to eat) in the last 1 month?** | \|___\|___\| | \|___\|___\|___\| days |
| fat6  fatdu6 | **12** | **Do you feel tired most of the time?** | \|___\|___\| | \|___\|___\|___\| days |
| osxs61 | **13** | **Do you have any other symptoms?**  If yes, specify: | \|___\|___\| | \|___\|___\|___\| days |
| osxs62 | **14** | **Do you have any other symptoms?**  If yes, specify: | \|___\|___\| | \|___\|___\|___\| days |
| Preg | **15** | **Are you pregnant?** | \|___\|___\| |  |
| bslnsxs | **16** | **Has the study participant had any symptoms suggestive of TB (i.e. any symptoms 1-13)?**  00=No; 01=Yes; | \|___\|___\| | bslnsxs |
|  |  | **If female give a cup and obtain urine specimen for urine pregnancy test**  **If yes give sputum cup and instructions for sputum collection. Also give a CXR request form. Do not give form if pregnant** |  |  |

**“I will now talk to you about tuberculosis.”**

| TBdx6 | **17** | **Have you been diagnosed with TB in the last 6 months?**  00=No; 01=Yes; 99=Don’t know | \|___\|___\| |
| --- | --- | --- | --- |
|  |  | **If no skip to Question # 25**  **if yes ask for TB treatment card . If patient does not have TB treatment card ask for name of clinic where TB treatment was started in order to abstract information** |  |
| TBrxnow | **18** | **Are you on treatment for TB**  01=Yes; 02=No; 98=missing CRF data; 99=D/K | \|___\|___\| |
| TBrxdte | **19** | **When did you start treatment? (**dd/mmm/yyyy)01/mm/yyyy if day not 01/111/1119=if day, month and year not known  01/1111/yyyy if day and month are not known  01/mmm/yyyy if day is not known  1/11/1117=N/A (not on treatment); 11/11/1118=missing CRF data; 11/11/1119=D/K | \|___\|___\|/\|___\|___\|/\|___\|___\|___\|___\| |
| TBepitype | **20** | **Episode type**  01 = New; 02= Re-treatment 03= MDRTB  09= unknown/no record | \|___\|___\| |
| TBsite | **21** | **Site of TB diagnosed**  01 = Pulmonary 02= Extra-pulmonary 0 3= both/disseminated 98= unknown ; 99= Don’t know/ No record | \|___\|___\| |
| TBpulm | **22** | **If pulmonary, specify smear status**  01 = smear positive 02= smear negative, culture positive  0 3= smear negative, culture negative ; 99= Don’t know/ No record | \|___\|___\| |
| TBext | **23** | **If Extrapulmonary, specify site**  01 = lymph nodes ; 02=meningeal; 03=pleural; 04=pericardial; 05= abdominal; 06=military; 07=disseminated TB; 08= skeletal TB ;  09=other ; 99=Don’t Know/ No record | \|___\|___\| |
| TBrxcom | **24** | **Have you completed TB treatment in the last 6 months**  01=Yes; 02=No; 98=missing CRF data; 99=D/K | \|___\|___\| |
| TBrxout | **25** | **If yes what was outcome of TB treatment**  01 = cured; 02=treatment completed; 03=defaulted; 04=MDRTB | \|___\|___\| |
| con6wk | **26** | **Has someone you know been diagnosed with TB in the last 6 months?**  0=No; 1=Yes; 9=Don’t know | \|___\|___\| |
| TBlv6w | **27** | **Has someone you live with been diagnosed with TB in the last 6 weeks?**  0=No; 1=Yes; 9=Don’t know | \|___\| |

**“I will now talk to you about HIV and medications.”**

| cd46m | **28** | **Have you had a CD4 count done in the last 6 months**  00=No; 01=Yes; 99=Don’t know | \|___\|___\| |
| --- | --- | --- | --- |
| cd4dte | **29** | **Date of most recent CD4 count (**dd/mm/yyyy)  01/111/1119=if day, month and year not known  01/1111/yyyy if day and month are not known  01/mmm/yyyy if day is not known  11/11/1117=N/A (not on treatment); 11/11/1118=missing CRF data; 11/11/1119=D/K | \|___\|___\|/\|___\|___\|/\|___\|___\|___\|___\| |
| cd4res | **30** | **What was the result of your most recent CD4 count**  Give results in cells/µl  9777=N/A (never had a test done); 9999=Don’t know | \|___\|___\|___\|___\| |
| Ipt6m | **31** | **Have you started isoniazid for IPT since we last saw you**  00=No; 01=Yes; 97= on IPT at enrolment ; 99=Don’t know | \|___\|___\| |
| Cpt6m | **32** | **Have you started cotrimoxazole (Bactrim) since we last saw you**  00=No; 01=Yes; 97= on CPTT at enrolment ; 99=Don’t know | \|___\|___\| |
| Art6m | **33** | **Have you started taking ARV drugs since we last saw you**  00=No; 01=Yes; 97= on APT at enrolment ; 99=Don’t know | \|___\|___\| |
|  |  | **If started on ART please enter ART clinic number/ folder number**  **Into the locator form to allow verification of data.**  **If NOT started on ART and CD4 is less than the threshold for starting ART, refer to HIV clinic for ART.** |  |
| Ill6m | **34** | **Have you been ill in the last 6 months**  00=No; 01=Yes; 97= on APT at enrolment ; 99=Don’t know | \|___\|___\| |
| Hosp6m | **35** | **Have you been hospitalized since we last saw you**  00=No; 01=Yes; 99=Don’t know | \|___\|___\| |
| con6m | **36** | **Has someone you know been diagnosed with TB in the last 6months?**  00=No;0 1=Yes;0 9=Don’t know | \|___\|___\| |
| TBlv6m | **37** | **Has someone you live with been diagnosed with TB in the last 6 weeks?**  00=No; 01=Yes; 09=Don’t know | \|___\|___\| |

**“I will now weigh and measure you.”**

| wt6 | **38** | **Weight today in kilograms:** | \|___\|___\|\|___\|**.**\|___\| |
| --- | --- | --- | --- |
| wtls6 | **39** | **Has the study participant lost weight since the enrollment visit?**  00=No; 01=Yes; 09=Don’t know | \|___\|___\| |
| ht6 | **40** | **Height today in centimeters:** | \|___\|___\|\|___\|**.**\|___\| |

**I will now give you a date on which you need to return for a follow up visit”.**

| addr | **40** | **Has your address changed since we last saw you?**  00=No; 01=Yes; 09=Don’t know | \|___\|___\| |
| --- | --- | --- | --- |
| tel | **41** | **Has your telephone numbers changed since we last saw you:**  00=No; 01=Yes; 09=Don’t know | \|___\|___\| |
|  |  | **If yes please update contact details in the locator form started** |  |
| dtevis | **42** | **Date of next follow up visit** | \|___\|___\|/\|___\|___\|___\|/\|___\|___\|___\|___\| |

Completed by |___||___| Verified by |___||___| First entry: |___||___| Double entry|___||___|
